# Complicated Clinical Course of a Patient with Multivisceral Cystic Echinococcosis Requiring Extensive Surgical and Medical Treatment

**DOI:** 10.3390/jcm12175596

**Published:** 2023-08-27

**Authors:** Gabriela Loredana Popa, Alexandru Cosmin Popa, Bogdan Mastalier, Carmen Michaela Crețu, Mircea Ioan Popa

**Affiliations:** 1 Department of Microbiology, Faculty of Dentistry, Carol Davila University of Medicine and Pharmacy, 020021 Bucharest, Romania; 2 Parasitic Disease Department, Colentina Clinical Hospital, 020125 Bucharest, Romania; michaelacarmen.cretu@gmail.com; 3 Departament of Surgery, Faculty of Medicine, Carol Davila University of Medicine and Pharmacy, 020021 Bucharest, Romania; cosmin.popa@umfcd.ro (A.C.P.); bogdan.mastalier@umfcd.ro (B.M.); 4 Surgery Department, Colentina Clinical Hospital, 020125 Bucharest, Romania; 5 Department of Parasitology, Faculty of Medicine, Carol Davila University of Medicine and Pharmacy, 020021 Bucharest, Romania; 6 Department of Microbiology II, Faculty of Medicine, Carol Davila University of Medicine and Pharmacy, 020021 Bucharest, Romania; mircea.ioan.popa@umfcd.ro; 7 Cantacuzino National Military Medical Institute for Research and Development, 050096 Bucharest, Romania

**Keywords:** multivisceral cystic echinococcosis, rare location, extensive follow-up

## Abstract

Cystic echinococcosis is an often-overlooked condition that otherwise negatively impacts both the individual and the community, prompting major public health concerns. Early diagnosis and treatment, as well as collaboration between specialties including surgery and parasitology, are essential for avoiding complications and disease relapse. To better illustrate this, we present the case of an elderly person with a rare localization of the disease at the muscular level. The patient underwent numerous surgical interventions, and received multiple courses of antiparasitic treatment over the course of 40 years as a result of the multivisceral dissemination of the parasite.

## 1. Introduction

Cystic echinococcosis (CE), recognized in the medical literature as a disease frequently encountered in the countryside [1,2,3], has come to affect the urban population of our country, which is presumably related to the large number of stray dogs, which are the definitive hosts for *Echinococcus granulosus* [4,5,6].

This disease can be asymptomatic for a long time, and is consequently often only diagnosed at a late stage, when the hydatid cyst has grown to a large size. There is an increased risk of complications when the cyst has grown to a considerable size, including cyst rupture with or without anaphylactic shock, multivisceral dissemination, cyst fissure with cavity infection and secondary abscess, development of sepsis, etc., all of which are circumstances associated with reserved prognosis [7,8].

For CE, consensus has been reached on an image-based, stage-specific approach, which is helpful for choosing one of the following options: (1) percutaneous treatment; (2) surgery; (3) anti-infective drug treatment; or (4) watch and wait [7].

The results of a systematic review and meta-analysis revealed a correlation between the cyst stage of hepatic CE and the results of serological tests, with the highest sensitivity being found in the presence of CE2 and CE3 and the lowest in the presence of inactive cysts [9]. These findings show that, both in the clinical environment and in the context of research projects, it is imperative to define and take into consideration cyst staging when analyzing the serological data of individuals with hepatic CE [4,7,9].

Recent findings support the hypothesis that chitosan nanoparticles have the potential to be a safe and efficient candidate scolicidal agent at very low concentrations and over a wide range of exposure times [10]. To assess the effectiveness of chitosan nanoparticles prior to therapeutic application, more in vivo investigations are recommended [10].

We conducted an evaluation of worldwide databases in order to assess the existing information on multivisceral cystic echinococcosis. For this reason, we initially looked for medical papers related to the keywords “hydatidosis” and “echinococcosis” in combination with “multivisceral” in PubMed/Medline. Despite not specifying the year of publication, we discovered only 33 publications, of which 4 were fundamental laboratory studies, 14 were conference papers, and the rest were case reports. Most frequently, the authors reported on topics such as multivisceral cystic echinococosis in children [11] and the occurrence of subcutaneous hydatid cysts in the greater context of multivisceral CE [8], as well as rare dissemination sites, such as the heart or the pelvis, or in the context of spinal cord compression syndromes [12,13,14,15]. A patient with multivisceral CE affecting the left infratemporal fossa, heart, liver, pancreas, abdomen and pelvic cavity was treated with local surgery, due to the unusual localization, and chemotherapy to lower the risk of local recurrences, as well as to address other infection sites [16]. A similar attitude was registered in a case of multivisceral CE with spontaneous rupture of a splenic hydatid cyst complicated by anaphylaxis [17].

We also looked at the data provided by Clarivate Analytics to see whether other databases offered more articles on multivisceral cystic echinococcosis (webofscience.com, accessed on 29 June 2023). We identified seven articles, four of which were very similar to those published in PubMed, one without relevance to the studied domain, and the others treating osseous CE, with multivisceral implications [18,19,20]. We estimate that a more exhaustive review would probably reveal more such studies.

Taking this into account, as well as the fact that multivisceral CE is a difficult and uncommon disease with limited therapeutic options, we aim to report the case of a female patient, which we consider will be of value in increasing both the theoretical and practical knowledge of this subject.

## 2. Case Report

The patient, a 79-year-old female, has been on the records of our clinic for 13 years, undergoing monitoring and treatment of multivisceral cystic echinococcosis.

The most important elements of the patient’s personal history at the time of presentation to our clinic were six surgical procedures for: retroperitoneal hydatid cyst in the right lumbar region, operated in 1974, reoperation for lumbar relapse in 1976, reoperation for secondary retroperitoneal echinococcosis in 1984, right kidney hydatid cyst, operated in 1987, relapse of right kidney hydatid cyst, operated in 1990, and relapse of multiple retroperitoneal CE, operated in 1995 (Supplementary Figures S1–S3).

Between 1974 and November 2000, the patient was not receiving any specific antiparasitic treatment. On 17 November 2000, she presented to the Parasitic Disease Department of Colentina Hospital. On admission, the patient had elevated inflammatory markers and mild anemia. The ELISA test for specific anti-*Echinococcus granulosus* IgG antibodies (Ab) showed a value of 5.9 (minimal positive value = 1.1).

Cardiac, thoracic and pulmonary radiographic examination showed two masses. The mass located at the posterior base of the right lung had the characteristics of a cyst.

Abdominal ultrasonography highlighted the presence of multiple cystic masses (summing to 15/10 cm) in the renal space with extension to the right flank, which does not allow viewing of the right kidney structure. 

The abdominal and pelvic CT scan confirmed the presence of multiple hydatid cysts, distributed on the projection area of the right retroperitoneal space, beginning with the retrocrural space, extended caudally towards the right pre-psoas muscle space as well as towards the level of the right kidney.

Combined antiparasitic treatment was started (Albendazole 10 mg/kg/day and Praziquantel 25 mg/kg/day) in 30-day courses, separated by 2-week medication-free intervals, all throughout 2001.

The patient returned to our clinic in February 2002 with fever, chills, sweating and purulent cough. The base of the right lung presented rales on auscultation and dullness on percussion. Routine laboratory tests showed: erythrocyte sedimentation rate (ESR) 106 mm/h, fibrinogen 967 mg/dL, leukocyte count 11.000/mmc (76% neutrophils), gamma glutamyl transpeptidase 347 UI/L, alkaline phosphatase 263 UI/L.

The chest radiograph showed a well-defined pseudo-tumoral mass at the base of the right hemithorax, with air-fluid level inside, leading to the suspicion of an infected, ruptured hydatid cyst.

The abdominal ultrasonography revealed a huge mass of mixed consistency, under the liver, probably situated in the retroperitoneal area, 10/6 cm in size, coming into contact with the right branch of the portal vein (without invading it) and with the inferior vena cava.

Parenteral treatment with Metronidazole was started, 1 g every 12 h and Cefotaxime 1 g every 8 h, alongside the treatment with Albendazole. The patient was transferred to the Surgery Department for surgical intervention.

Intraoperative examination revealed multiple visceral–visceral and parieto–visceral adhesions, requiring surgical lysis. Partial peri-cystectomy, draining of the infected cyst, and antiseptic lavage were performed for the retroperitoneal hydatid cyst located in the right iliac region. An additional hydatid cyst in the VII-VIII liver segments was noticed, penetrating the diaphragm and compressing the right lung. The cyst removal procedure entailed drainage, antiseptic lavage, antegrade cholecystectomy and partial omentectomy. Overall, surgical treatment included double drainage for the liver hydatid cyst, drainage for the right iliac region hydatid cyst, peritoneal lavage, subhepatic and Douglas pouch, as well as subcutaneous drainage.

The patient followed the antiparasitic treatment until June 2002. At that time, she presented for her previously scheduled evaluation (routine laboratory tests, normal values; hydatid ELISA, 4.4; an entrapped pleural effusion in the right hemithorax was seen at the radiographic examination; no cystic images in the upper abdomen at the ultrasonography).

In August 2002 she presented with fever and hemoptysis. At the beginning of September, she was admitted to the Thoracic Surgery Department, where radiographic examination revealed a large pleural effusion, located in the base of the right lung. The lateral thoracic radiograph revealed an opaque oval mass, located in the right upper third of the lung, near the posterior wall. Abdominal ultrasonography showed the following: a relatively homogenous liver, absence of the gallbladder, a regular, homogeneous pancreas, normal-sized and homogeneous spleen, irregular contour and disorganized aspect of the right kidney, and normal left kidney. Thoracic and abdominal CT imaging revealed multiple hydatid cysts in various locations (pleura, lungs, diaphragm, retroperitoneal area, and liver).

For the right lung cystic echinococcosis, the surgeons used the Fracer–Gurd decortication technique. The procedure was complicated by a right posterior base hydatid empyema. In addition, the retroperitoneal cyst that fused trans-diaphragmatically behind the liver was evacuated. Pericystectomy was performed with transparietal and diaphragm drainage.

The patient underwent three postoperative antiparasitic treatment courses, then she presented again for her usual check-up. The ultrasonography confirmed the absence of any abdominal hydatid cysts and a 3.5 cm residual liver cavity. The right lung showed retraction and sequel pachypleuritis.

The patient finished her medical treatment in February 2003. She was regularly monitored twice a year in the first two years, then annually. In May 2005, the antibody titer was 1.2, compared to a positive minimum of 1.1.

**Figure 1:**
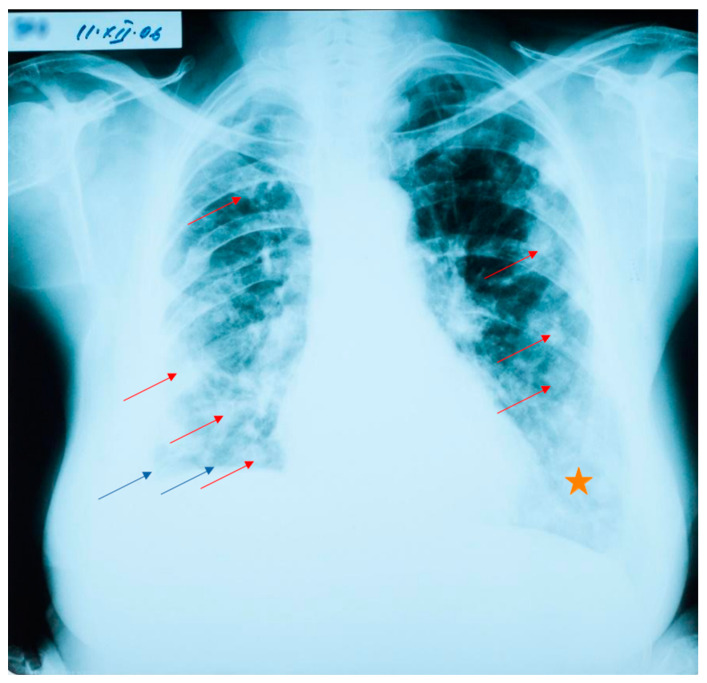
Lung radiograph with numerous bilaterally disseminated opacities. Blue arrows show the elevation of the right diaphragm, and the blunting of the lateral costo-diaphragmatic angle. Red arrows point to round opacities disseminated bilaterally (not all are marked, as to not overcrowd the image). The orange star denotes an area with a diffuse para-cardiac opacity.

At her check-up in October 2006, the thoracic radiograph raised the suspicion of lung metastases from a cancer of unknown location (numerous 1–2 cm opacities, with bilateral lung dissemination) (Figure 1). 

**Figure 2:**
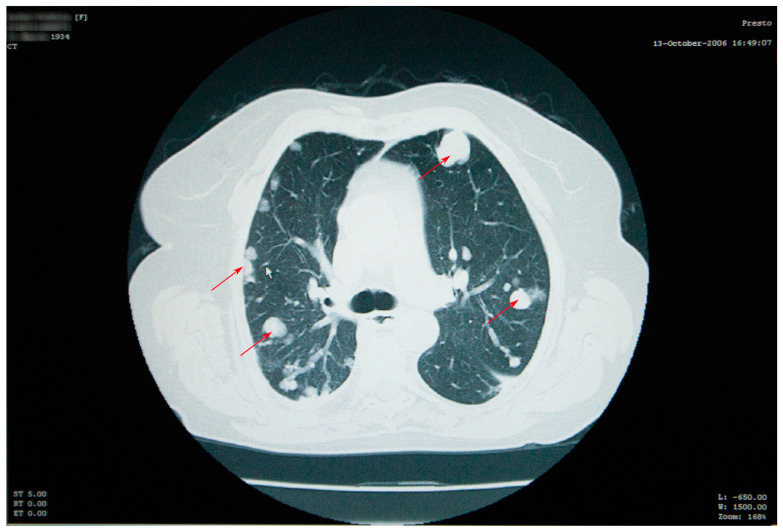
Thoracic CT scan showing numerous thoracic masses (red arrows), located bilaterally, with different consistencies, and bilateral pachypleuritis.

Thoracic and abdominal CT scans were performed, which revealed numerous thoracic masses, located in both the lungs and the pleura, round or oval in shape, centimetric or millimetric in size, with different consistencies, some liquid with calcifications, some condensed, sometimes clustering, as well as bilateral pachypleuritis, more pronounced on the right side (Figure 2). 

**Figure 3:**
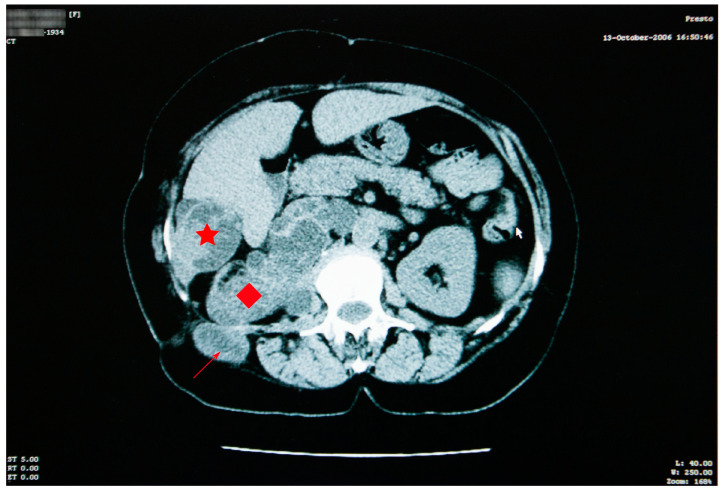
Abdominal CT scan showing multiple cysts, confluent in the right kidney space (red diamond), invading the retroperitoneal area, in the sixth right liver segment (star) and in the right iliac fossa and right paravertebral area (arrow).

Abdominal imaging revealed multiple cysts, with lots of septa, confluent in the right kidney space, invading the retroperitoneal area down into the right pelvic region, situated between the aorta and the vena cava. Similar types of damage were visible in the sixth right liver segment, as well as the right iliac fossa, and the right paravertebral region (Figure 3). 

The right kidney could not be visualized. The psoas muscle, along with the spaces between intestinal loops, had been infiltrated by the same sort of septate cystic masses. A somewhat larger mass, with a size of 45/33 mm, was observed adjacent to the bowel on the right side. The specific serology was intensely positive.

Classical antiparasitic treatment with Albendazole was instituted again. After eight therapeutic courses, in 2007, the abdominal CT scan revealed no changes; however, the thoracic CT indicated small improvements, with the cysts having a mixed solid and liquid nature (and a tendency towards densification and the elimination of clustering).

The antiparasitic treatment was continued.

**Figure 4:**
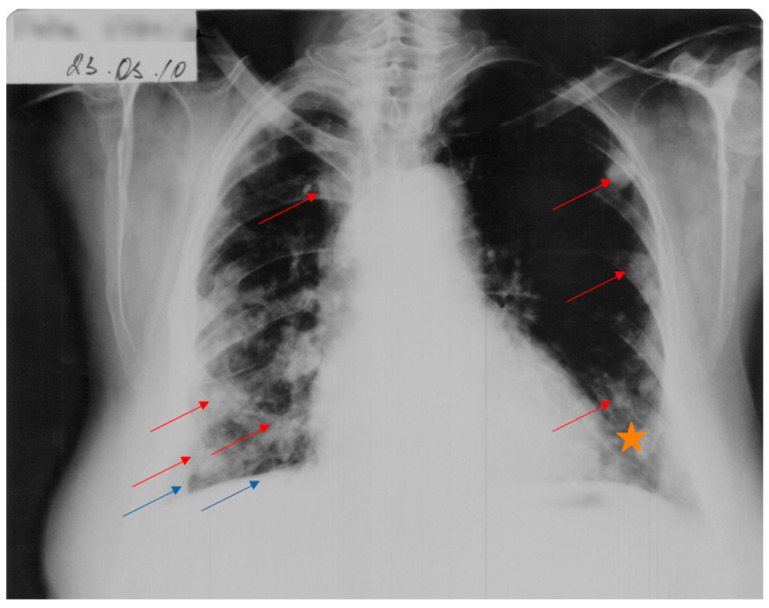
Cardio-thoracic radiograph showing a favorable evolution of the cysts (red arrows) and diffuse para-cardiac opacity (star) under antiparasitic treatment. Persistence of right-lung retraction can be observed (blue arrows).

A favorable evolution of the cysts under antiparasitic treatment was observed in October 2009 and March 2010 (Figure 4). 

**Figure 5:**
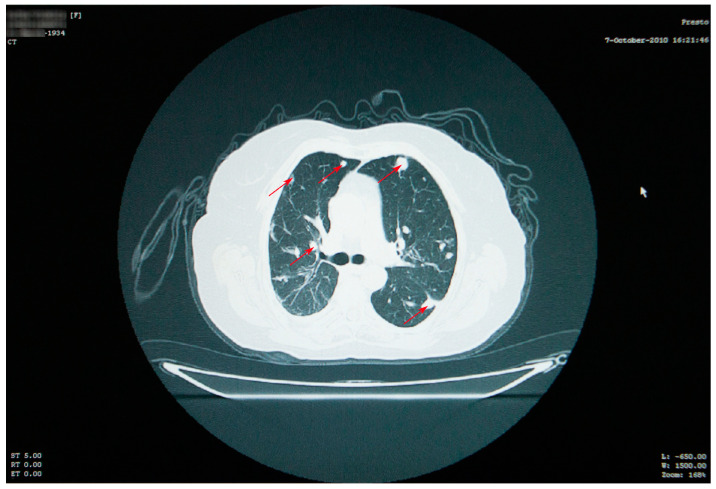
Thoracic CT showing a reduction in the appearance and dimensions of the cysts (red arrows), which is associated with favorable evolution under treatment.

Multiple solid lung masses were described, some with a nodular appearance, some spiculated extensions, some with well-defined borders, and in the approximately same position as the nodular masses from 2006, although obviously diminished in size and with a global change in terms of appearance and contour (Figure 5). The number of masses was unchanged since the previous CT scan; the posterior and right lateral thoracic fibrotic pleural alterations remained; the right pachy-pleuritis was more visible in the right posterior and superior areas.

**Figure 6:**
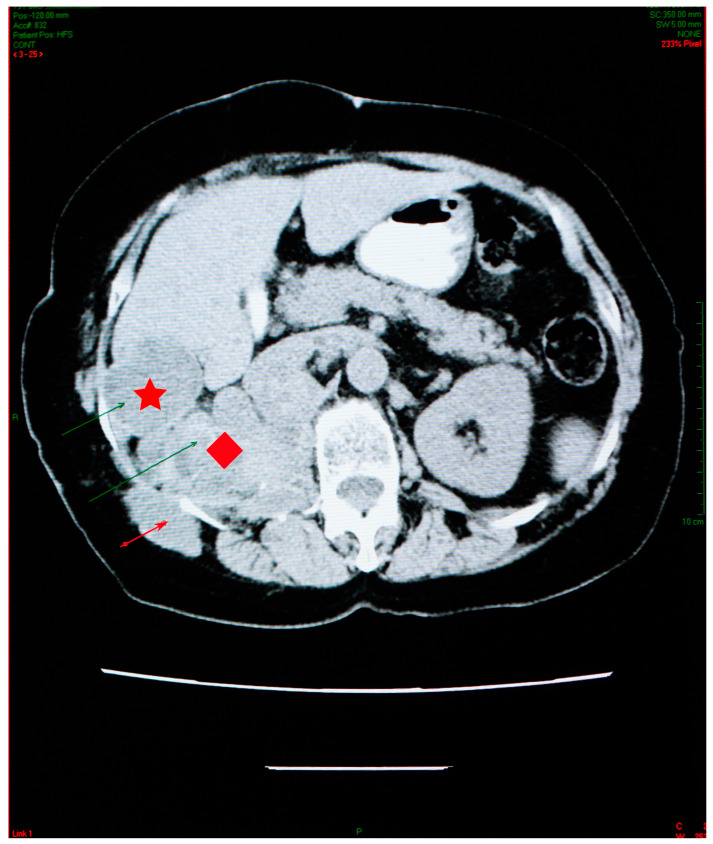
Abdominal CT shows reduction in the aspect and dimensions of the cysts—right kidney space (red diamond), the sixth right liver segment (star) and in the right iliac fossa and right paravertebral area (arrow)—associated with favorable evolution under treatment.

The abdominal CT scan showed multiple spontaneous hypodense masses with hyperdense borders, occupying the entire kidney space, with no evidence for the right kidney (Figure 6). 

The maximum axial dimension of the above-mentioned lesions was 10 cm, which is considerably less than what was recorded in 2006. In addition, this conglomerate of hypodense images was remarked to have increased in overall density. The conglomerate presented cranial and caudal extension, starting just beneath the diaphragm and ending right under the plane of the renal artery, with a close anatomic relation to the right diaphragm pillar, right hemidiaphragm, and right psoas muscle. The spontaneous hypodense, heterogenous mass, with an axial maximum diameter of 46 mm, located in the VI liver segment, appeared to be reduced in comparison to the 2006 examination.

Between 2009 and 2013, the patient presented twice a year to her regular check-ups. We observed the lung injuries to possess stationary and condensed characteristics, whereas the liver mass remained the same size but, had nearly totally condensed. The renal space conglomerate decreased slightly in size, having a more condensed image, with the persistence of small areas of liquid densities inside.

We noticed the presence of the inflammatory syndrome and that the titer of the antibodies against *Echinococcus granulosus* reached a value a value of 1.8. We must additionally emphasize that the patient underwent further surgical procedures, including a postoperative hernia repair, a right knee prosthesis, and the placement of a permanent cardiac stimulator (first-degree atrioventricular block, intermittent second-degree atrioventricular block).

## 3. Discussion

According to the experience of the Colentina Hospital’s Parasitology Department (Rădulescu et al., unpublished), postoperative recurrences occur in 20.4% of cases after 4 years, as was the case in the current report. The only difference is that participants in our 2004 study had not undergone postoperative antiparasitic therapy. Although the patient had received three courses of antiparasitic therapy after the surgical intervention in September 2002, the length of her treatment was shorter than is now recommended (at that time two or three postoperative courses were considered to be sufficient) [4,7,21,22,23,24,25].

In this particular case, which presents the clinical course of a secondary, disseminated, cystic echinococcosis, we noticed that the disease was much more severe and disabling compared to the primary hydatid cyst, when accounting for the number of cysts, their locations, and any complications (ruptured and infected hydatid cysts, secondary hydatid pleural effusion, multivisceral dissemination, disorganized right kidney structure, toxic status, and risk of sepsis). We reviewed the papers found in the literature related to primary multivisceral CE in the Introduction. Evaluating Clarivate Analytics databases, we identified only 15 articles addressing the subject of secondary multivisceral hydatid disease, thus strengthening the usefulness of this case report.

The particularity of our case consists in the rare primary location (right lumbar, retroperitoneal), which was secondary to intraoperative protoscolices dissemination, as well as the fact that the patient underwent eight surgical interventions as a result of the multivisceral disease.

Surgical treatment is considered to ensure healing in the case of CE, because it completely removes the cause of the disease [26], but medical treatment must be associated with surgical therapy in order to prevent hydatid recurrence. When patients with CE cannot be operated on—either due to contraindications (e.g., associated diseases) or the locations being difficult to reach, the cysts being very small, or the presence of numerous tiny cysts—the only option is medical therapy [7,16,21,22,23,24,25].

In echinococcosis caused by *E. granulosus*, the fibrous capsule that surrounds the cyst may prevent antiparasitic treatment from entering the tapeworm tissues, and antiparasitic drugs can be diluted in the hydatid fluids. Thus, effective medical chemotherapy is a lengthy process, explaining the need for repeated courses, over a period of 2–3 years, depending on the number, size, viability, and location of cysts, and the patient’s age. Single hydatid cysts respond better to antiparasitic treatment than do multiple cysts (which produce recurrences in 25% of cases) [21,22,23,25].

The patient was recommended to take Albendazole after a fat-rich meal [21,27,28]. It was not possible for us to check the patient’s compliance. It is possible that non-compliance with the recommendation contributed to the relatively poor overall therapeutic outcome.

We learned that giving antiparasitic medication for only three months was insufficient to avoid relapses in operated patients, especially if the surgical interventions were laborious, or being performed on larger cysts or multiple determinations [7,16,23].

Abdominal ultrasonography is very useful, but sometimes insufficient for the monitoring of situations that involve several abdominal surgical procedures, as well as cases involving retroperitoneal CE. The post-surgical residual cavity, visible after more than 18 months, strengthens the possibility of recurrence, as has been documented previously in the literature [29,30]. Computed tomography scanning has many advantages for the diagnosis of multivisceral CE and postoperative surveillance [16,25]. It is our opinion that patients at high risk of relapse should be annually monitored though CT imaging, with contrast, and that ultrasonography should be used exclusively in control investigations every three months.

The existing serological diagnostics, which use native or recombinant antigens derived from protoscolices, have a significant limitation in that they cannot differentiate between past infections and recent infections or relapses [9,31]. Because of the activation of immunological memory, IgG levels can remain positive for several years after surgery. In the case of reactivation/relapses, the TH2 levels rapidly increase, while TH1 levels and total IgE show a slow evolution trend [32].

During a three-year monitoring period, the evolution of the specific serology should lead to a gradual decline, and can be profoundly positive in the case of a fresh recurrence. Biannual patient monitoring was demonstrated to detect the occurrence of probable relapses and establish the need to introduce antiparasitic therapy when the hydatid cysts are still incipient (these respond more favorably than larger cysts).

The expertise of the Colentina Hospital’s Parasitology Clinic, as well as the hospital’s Surgery Clinic, emphasizes the importance of the interdisciplinary therapy required by some patients, such as the patient reported in this paper. As highlighted by a recent publication, medical and surgical care of *E. granulosus* infection is complex due to the slow development of the cysts and the disease’s wide range of possible complications [33]. It is worth noting that the patient described by the Italian team was of Romanian origin and had emigrated to Sardinia 23 years prior. This is especially important for medical and surgical teams operating in nations where the incidence of disease is particularly low.

## 4. Conclusions

Because of its acute (associated with complications) and chronic progression, cystic echinococcosis is not only a public health concern that is largely overlooked, it is also a major problem for individuals and health systems, with considerable expenditures and hazards [3,4,16,21,25].

In areas with high prevalence of echinococcosis, examination by full imaging is necessary for accurate diagnosis, especially in cases with atypical localization. Cases of multivisceral CE with atypical localization are very rare, but can have severe consequences and may even be fatal.

The regular and long-term follow-up of patients with CE is highly recommended [7]. In order to decrease the risks of recurrence, we emphasize the importance of preoperative and postoperative medical care [7,21,23]. Unfortunately, we did not have the material resources to genotype the cystic material, establish the parasite strain, or detect albendazole levels in the blood at the time.

In recent years, prolonged antiparasitic regimens (90 or 180 days, or even longer) have been recognized as being more effective in both the specialized literature and in our clinic [7,23]. The courses can be repeated depending on the particularities of each case (e.g., evolution of the hydatid cysts, albendazole tolerance, etc.). Each hydatid cyst evolves as an independent entity, showing distinct responses to specific treatment, with large, aged cysts with a thick outer membrane being more resistant [21].

Another potential limitation of this study is that pure 20–30% saline solution was not accessible during surgeries, and betadine was not utilized in surgical departments back then.

While the literature has discussed the effectiveness of serological tests primarily in the context of diagnostic and surgical cases, suggesting them to be of limited benefit during case follow-up, in this specific instance, each recurrence was accompanied by a considerable increase in serological results [25].

Long-term follow-up of CE patients is critical because, according to our data (Cretu et al., unpublished), the majority of post-surgery relapses occur during the first 3–6 years.

Because of the unique, diversified, and unexpected progression of CE, close collaboration between experts (i.e., parasitology/infectious diseases, surgery, laboratory, and imaging) is strongly encouraged [7]. Such a collaboration has been established and has been operating extremely effectively at the Colentina Teaching Hospital for over a decade, enabling therapy to be adjusted to the requirements and individual options of each patient.

## Data Availability

No new data were created or analyzed in this study. Data sharing is not applicable to this article.

## Supplementary Materials

The following supporting information can be downloaded at: https://www.mdpi.com/article/10.3390/jcm12175596/s1, Figure S1: Timeline of patient evolution from diagnosis of CE to presentation at the Parasitic Disease Department, Colentina Clinical Hospital (years 1974–2000), Figure S2: Timeline of patient evolution between 2000–2003, when the patient received concomitant medical and surgical treatment, Figure S3: Timeline of patient evolution between 2003–2010, when patient received further medical treatment.
