# Supplementary material for: Complicated Clinical Course of a Patient with Multivisceral Cystic Echinococcosis Requiring Extensive Surgical and Medical Treatment
Source: J Clin Med. 2023 Aug 27;12(17):5596. doi: 10.3390/jcm12175596 (PMC10488279; doi:10.3390/jcm12175596)
Supplement: Supplementary file 1 [file jcm-12-05596-s001.zip › jcm-2507833-supplementary.pptx]

## Slide 1
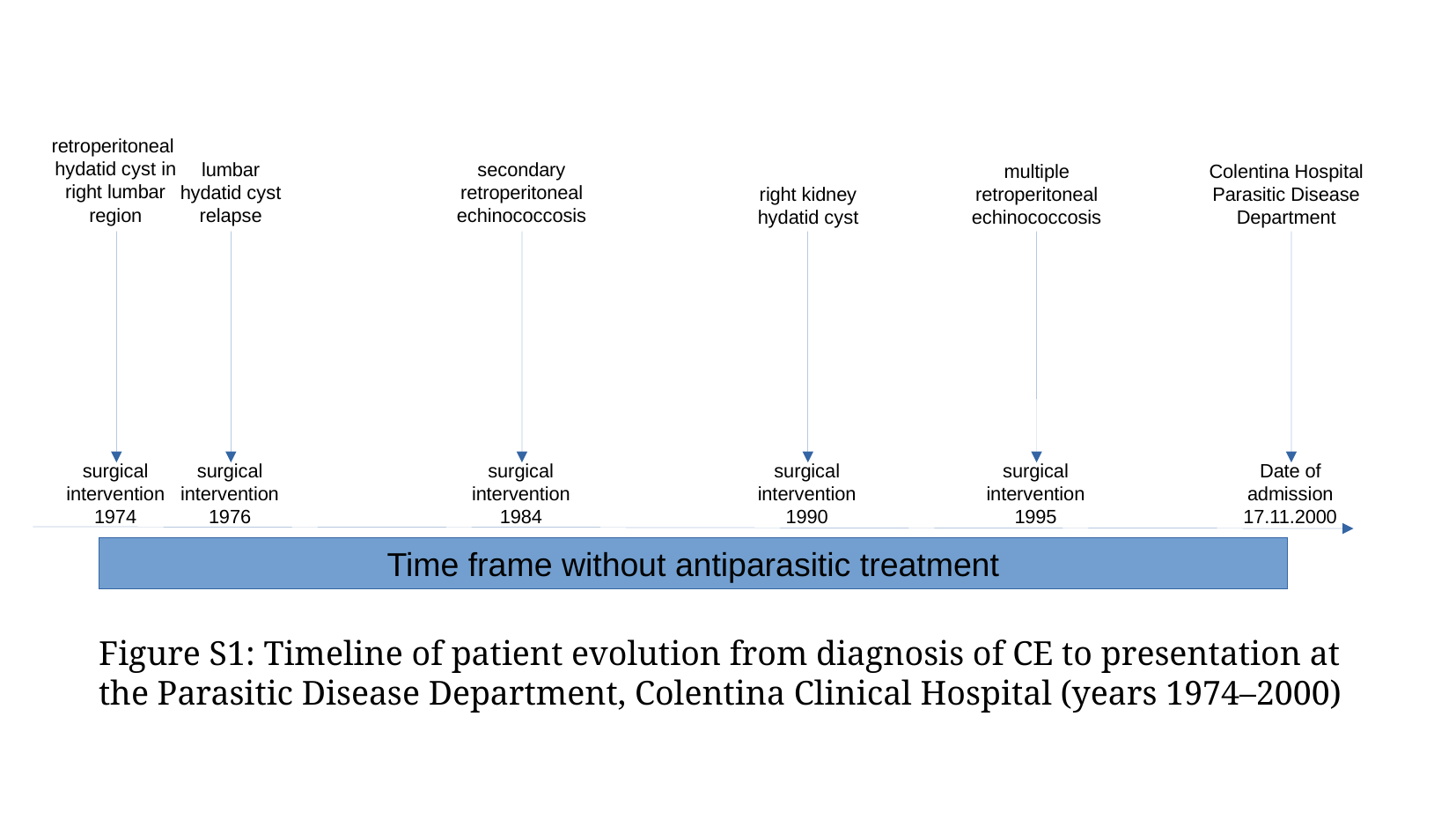

retroperitoneal
hydatid cyst in
right lumbar region
lumbar hydatid cyst relapse
secondary retroperitoneal echinococcosis
multiple retroperitoneal echinococcosis
Colentina Hospital Parasitic Disease Department
right kidney
hydatid cyst
surgical intervention
1974
surgical intervention
1976
surgical intervention
1984
surgical intervention
1990
surgical intervention
1995
Date of admission
17.11.2000
Time frame without antiparasitic treatment
Figure S1: Timeline of patient evolution from diagnosis of CE to presentation at the Parasitic Disease Department, Colentina Clinical Hospital (years 1974–2000)

## Slide 2
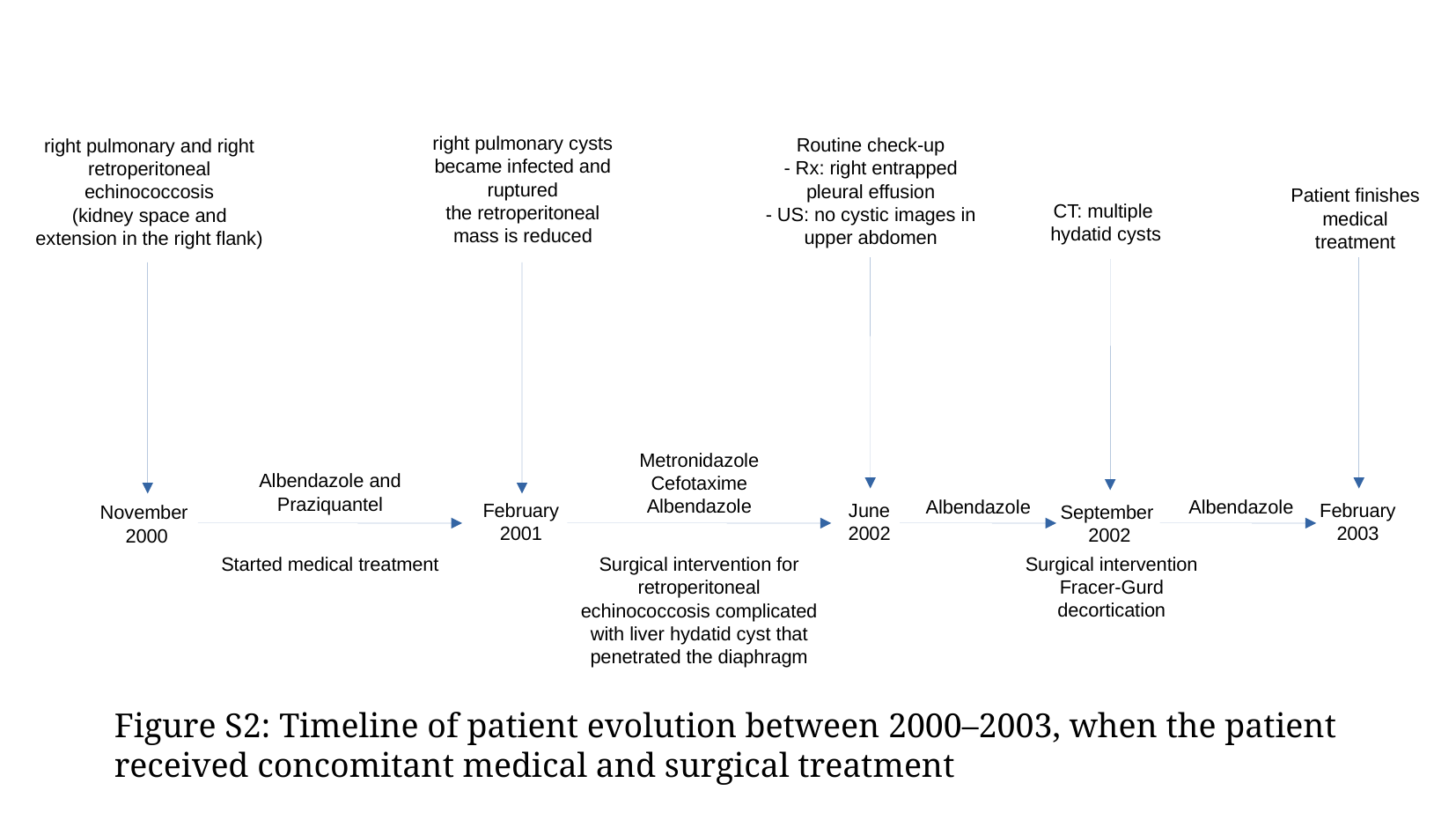

right pulmonary cysts became infected and ruptured
the retroperitoneal mass is reduced
Routine check-up
- Rx: right entrapped pleural effusion
- US: no cystic images in upper abdomen
right pulmonary and right retroperitoneal echinococcosis
(kidney space and extension in the right flank)
Patient finishes medical treatment
CT: multiple
hydatid cysts
Metronidazole
Cefotaxime
Albendazole
Albendazole and Praziquantel
Albendazole
Albendazole
February
2001
June
2002
February
2003
November
2000
September
2002
Started medical treatment
Surgical intervention for retroperitoneal echinococcosis complicated with liver hydatid cyst that penetrated the diaphragm
Surgical intervention
Fracer-Gurd decortication
Figure S2: Timeline of patient evolution between 2000–2003, when the patient received concomitant medical and surgical treatment

## Slide 3
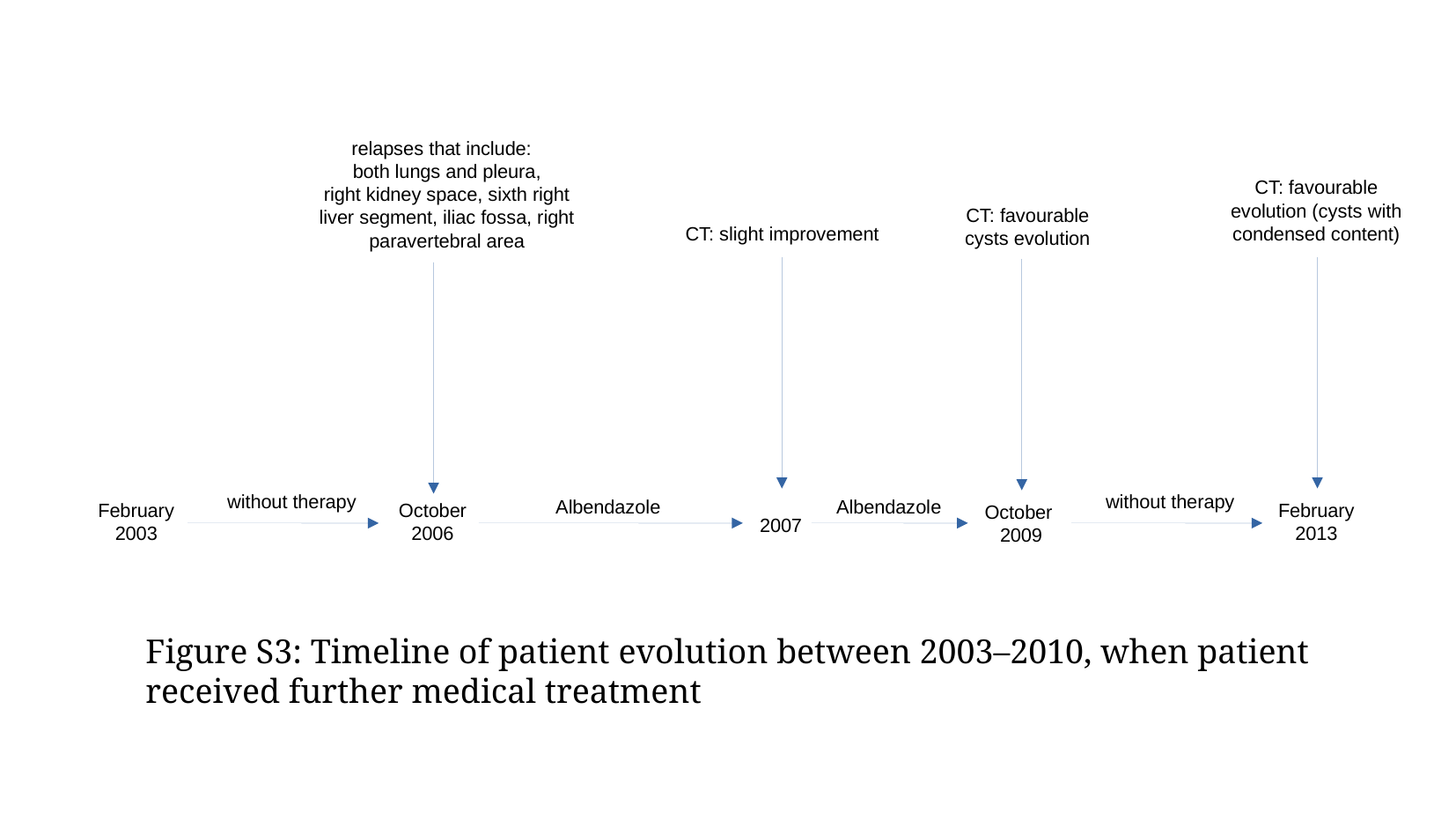

relapses that include:
both lungs and pleura,
right kidney space, sixth right liver segment, iliac fossa, right paravertebral area
CT: favourable evolution (cysts with condensed content)
CT: favourable cysts evolution
CT: slight improvement
without therapy
without therapy
Albendazole
Albendazole
February
2003
October
2006
February
2013
October
2009
2007
Figure S3: Timeline of patient evolution between 2003–2010, when patient received further medical treatment
